# Cost and pollution by the use of xylene in cervical cytology in four Peruvian hospitals

**DOI:** 10.12688/f1000research.52769.2

**Published:** 2022-04-21

**Authors:** Jeel Moya-Salazar, Richard Salazar-Hernández, Victor Rojas-Zumaran, Gloria Cruz-Gonzales, Hans Contreras-Pulache

**Affiliations:** 1 South America Center for Education and Research in Public Health, Universidad Norbert Wiener, Lima, +51, Peru; 2 Department of Pathology, Hospital Nacional Docente Madre Niño San Bartolomé, Lima, +51, Peru; 3 Department of Pathology, Hospital Nacional Guillermo Almenara Irigoyen, Lima, +51, Peru

**Keywords:** xylene, polycyclic aromatic hydrocarbon, cervical cancer, Pap test, pollution, Peru

## Abstract

**Background:** Cytological samples are cleared with xylene in two or three baths during a Pap test, however, this solvent has a high degree of toxicity, and being a controlled reagent infers high costs for its purchase and implications for environmental pollution. We estimated the impact of xylene during the Pap test in terms of the number of liters and cost of two baths of xylene, and also estimated the impact with three baths

**Methods: **This cross-sectional study was carried out in four hospitals of EsSalud in Peru in two stages. First, the analysis of the impact due to the use of two baths of xylene was conducted during the period 2015–2019, and second, the estimates were calculated based on the assumption of three baths of xylene for the years 2020–2025. The assumption was based on the recommendations of the 2018 EsSalud cytology guideline. The monthly amount of xylene was ~10 liters per bath/month and the cost per liter was estimated at 8.13 USD (27 soles).

**Results:** For the staining of 594,898 cytology tests, 7,848 liters of xylene were necessary, resulting in a cost of 60,861 USD (202,068 soles) during the period 2015–2019. The estimates showed a maximum assumption of 9,483 liters and 77,110 USD (256,040 soles) for the use of three baths of xylene in the four EsSalud hospitals (p = 0.0025) during the period 2020–2025.

**Conclusions:** We determined that there was a high economic impact of using xylene with two baths from 2015 to 2019 and a dramatic increase in costs with the possible use of three baths of xylene in the Pap test for the following five years.

## Introduction

Xylene (C _6_H _4_(CH _3_) _2_) is a polycyclic aromatic hydrocarbon (PAHs) part of the aromatic BTXs (benzene, toluene, and xylenes) obtained from petroleum, from the dry distillation of wood, and from coke gases. Xylene can have one of the three isomers of dimethylbenzene or a combination thereof, which are used as solvents (in fuel formulations, and glue in plastic model kits etc.). ^1^ In medical practice, xylene is used in cellular clarification in the final phase of tissue processing. ^2^^,^^3^

Since George Papanicolaou’s research began in 1914 at Weill Cornell University, these PAHs have been used as part of the Papanicolaou (Pap) stain to clear cells at the end (clearing) of the process. ^4^ The preliminary Pap protocol as well as the modifications that he made in 1953 and 1959 indicates the use of three xylene baths (~350 ml each) during the process. ^5^ These protocols, and several modifications ^6^^,^^7^ suggest the use of one or three xylene baths, causing high exposure to workers (as several hospitals do not have adequate protection barriers), bioaccumulation, and environmental pollution (due to the incorrect handling of these wastes). ^8^

Even though there are techniques for the biodegradation of xylene such as the use of iron and manganese from underground aquifers, the biofiltration of xylene by microorganisms adhered to a Nylon support, ^9^^,^^10^ and modifications of the Pap stain that avoid the use of xylene (and other pollutants), ^8^^,^^11^^,^^12^ laboratories in many countries continue using xylene as a cellular clarifier in two or three baths during the process. According to the standardized operating procedures of each cytology laboratory, these baths are replaced every thousand slides or once a week. ^12^

In this study, we aimed to estimate the impact of xylene during the Pap test in terms of number of liters and costs for two baths of xylene, and also estimated the impact with three baths for the next five years (2020–2025) following the 2018 Peruvian Social Security cytology guideline.

## Methods

### Study design, hospitals and inclusion criteria

**Figure 1.**
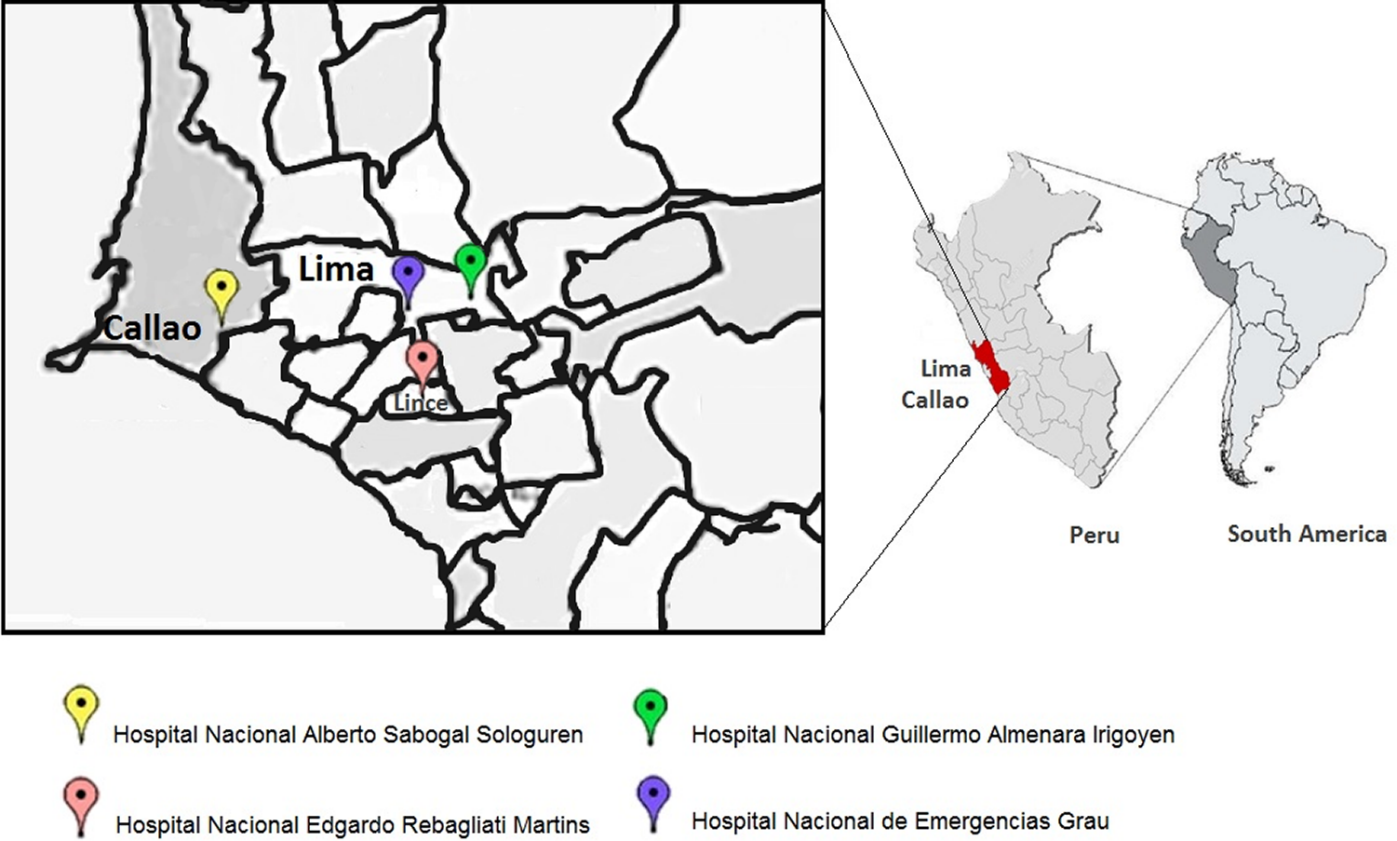
Location of the four EsSalud hospitals included in the study. Hospital Nacional Guillermo Almenara Irigoyen, Hospital Nacional Edgardo Rebagliati Martins, and Hospital Nacional de Emergencias Grau in Lima, and the Hospital Nacional Alberto Sabogal Sologuren in Callao province. Maps by ©Jeel Moya-Salazar.

This was a retrospective longitudinal study that used a model for estimating xylene prices and quantities through logistic regression and the Bayes test. This study was approved by the Institutional Review Board of the Universidad Norbert Wiener (Document UNW-N° 072-2020). The study was conducted in two stages with data on costs and quantities (in liters) of xylene used in cytology over a five-year period (2015–2019) in four hospitals ( Figure 1) of Social Security (EsSalud) of Peru. ^13^

o Hospital Nacional Guillermo Almenara Irigoyen (HNGAI)
o Hospital Nacional Edgardo Rebagliati Martins (HNERM)
o Hospital Nacional Alberto Sabogal Sologuren (HNASS)
o Hospital Nacional de Emergencias Grau (HNEG)

The EsSalud cytology guide, Chapter 9. Cervical cytology procedures in the laboratory: Section 9.5.1. Manual staining describes the process, which includes three xylene baths (10 minutes each) at the end of the staining procedure ( https://ww1.essalud.gob.pe/compendio/pdf/0000003706_pdf.pdf). ^14^^,^^15^ This new EsSalud cytology guideline aims to improve the Pap test procedure, seeking to standardize the process to reduce the incidence of cervical cancer. The report does not present a cost-effectiveness analysis and explains the necessary materials and equipment, but does not clearly describe the importance of changing each cytological sub-process. A brief English summary of the Pap stain process is available as supplemental material. ^14^

The inclusion criteria for this study were: i) EsSalud Hospital, ii) a hospital that performs a large number of Pap tests per year (approximately 25,000 Pap tests each), iii) a hospital that uses manual exfoliative cytology procedures. Hospitals belonging to the Ministry of Health, Armed Forces and Private Care Centers were excluded. These four tertiary hospitals are EsSalud's main reference hospitals for cytology and cancer screening in Lima and Peru.

### First and second stage

In the first stage we collected the data on xylene use for the period 2015–2019 in the four hospitals. ^13^ The amount of xylene used for exfoliative cytology was ~10 liters per bath/month (with two xylene-baths), ^14^^,^^15^ and the global cost (EsSalud) per liter of xylene was 8.13 US dollars (USD) per liter (27 soles). We estimated the number of cytology tests and the amount of xylene per year used as part of the routine Pap test, which follows the second protocol of Papanicolaou. ^4^

For the second stage, we followed the recommendations of the 2018 EsSalud cytology guidelines ^14^^,^^15^ that establishes the addition of a third xylene bath during Papanicolaou staining. We considered the use of three xylene baths for each cervical smear, estimating the monthly usage in each hospital. As in the previous stage, the cost of xylene was estimated.

### Cost analysis and pollution impact

We used the direct costs of purchasing xylene without considering the indirect costs of processing, logistics management and usage (man-hours) during the Pap test. The cost of one liter of xylene was determined according to the contract between the manufacturer and EsSalud, which is a standardized contract for all hospitals in Lima.

**Figure 2.**
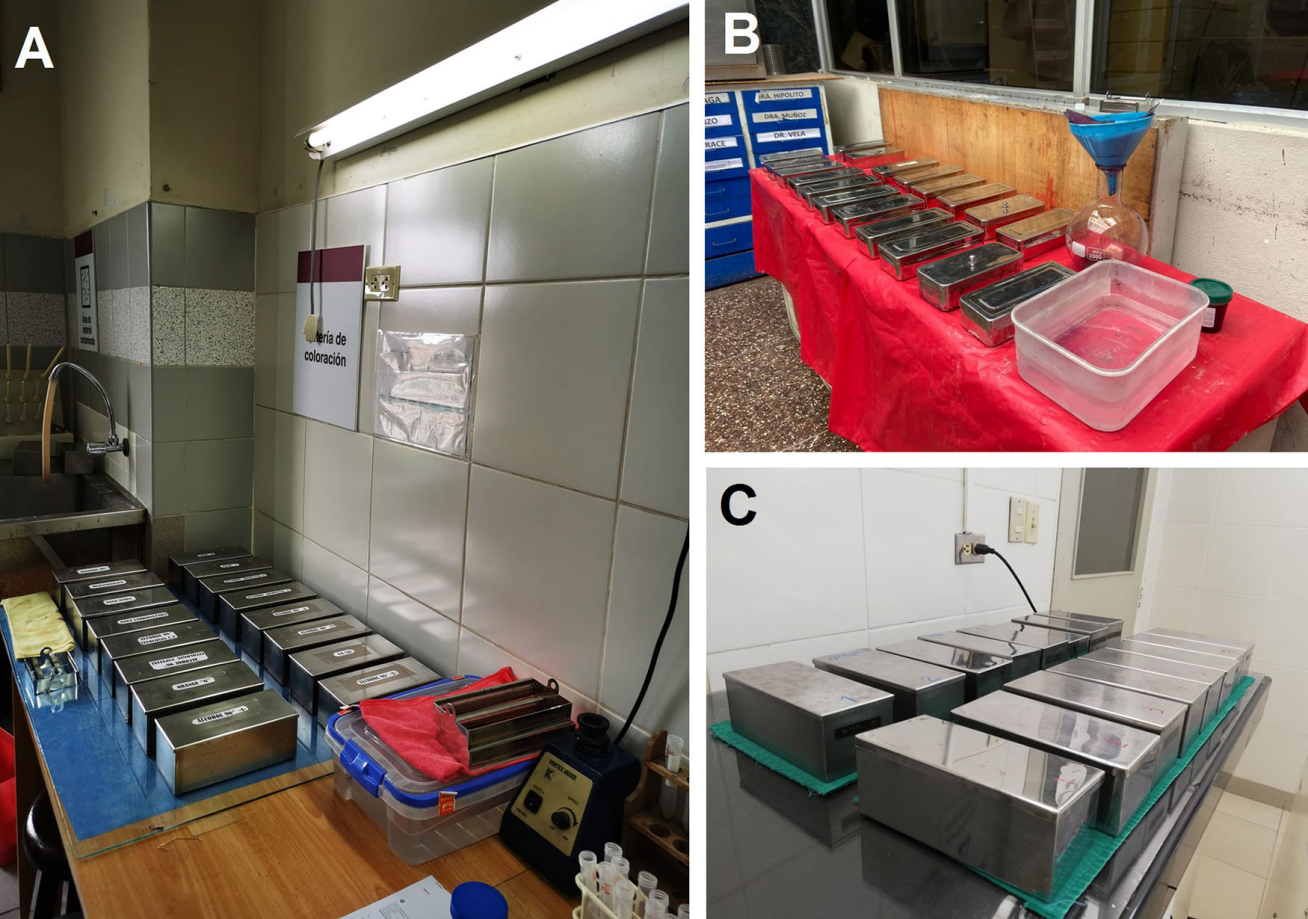
Biosafety conditions in cytology laboratories. (A) Guillermo Almenara Irigoyen National Hospital, (B) Hospital Nacional de Emergencias Grau, (C) Edgardo Rebagliati Martins National Hospital. Note that all staining centers are manual and are exempt from basic protection barriers such as the laminar flow chamber and air filtration systems.

Hence, we estimate the impact of pollution of xylene, initially by the amount of xylene used by the workers who were exposed daily to xylene under the biosafety conditions available in each hospital ( Figure 2). Then, we estimated the pollution load based on the bioaccumulation of xylene during use, because the waste collection cycle is once every two months (hospital accumulation process). The quantity of liters and the costs (in USD) for xylene were estimated only for the Pap tests (we exclude the amount of xylene used in histology).

In this sense, the monthly amount of xylene used in each hospital was estimated to establish the annual and the total cost (in liters). In the second stage, these costs and contamination estimates were made for the next five years (2020-2025) under the same parameters. The maximum and minimum amounts of xylene per year were used to estimate the costs and future quantities of xylene.

### Statistical analysis

Statistical analysis was performed with descriptive statistics and frequency measures. To estimate the cost, directly purchased xylene was used, with an estimated price of USD 8.13 (27 soles) per liter. In order to assess the impact of contamination (two baths for cytological clearance), the annual reagent usage and the annual xylene bioaccumulation rate of four hospitals in Lima were considered. To estimate the impact of pollution and direct costs (between 2020–2025), of using three xylene baths, multiple logistic regression with Bayesian analysis was used, with a p-value <0.05 and a 95% confidence interval (CI) as significant. We used IBM Statistical Package for the Social Sciences ( SPSS) v25.0 (Armork, US) for Linux for all data analysis.

## Results

**Figure 3.**
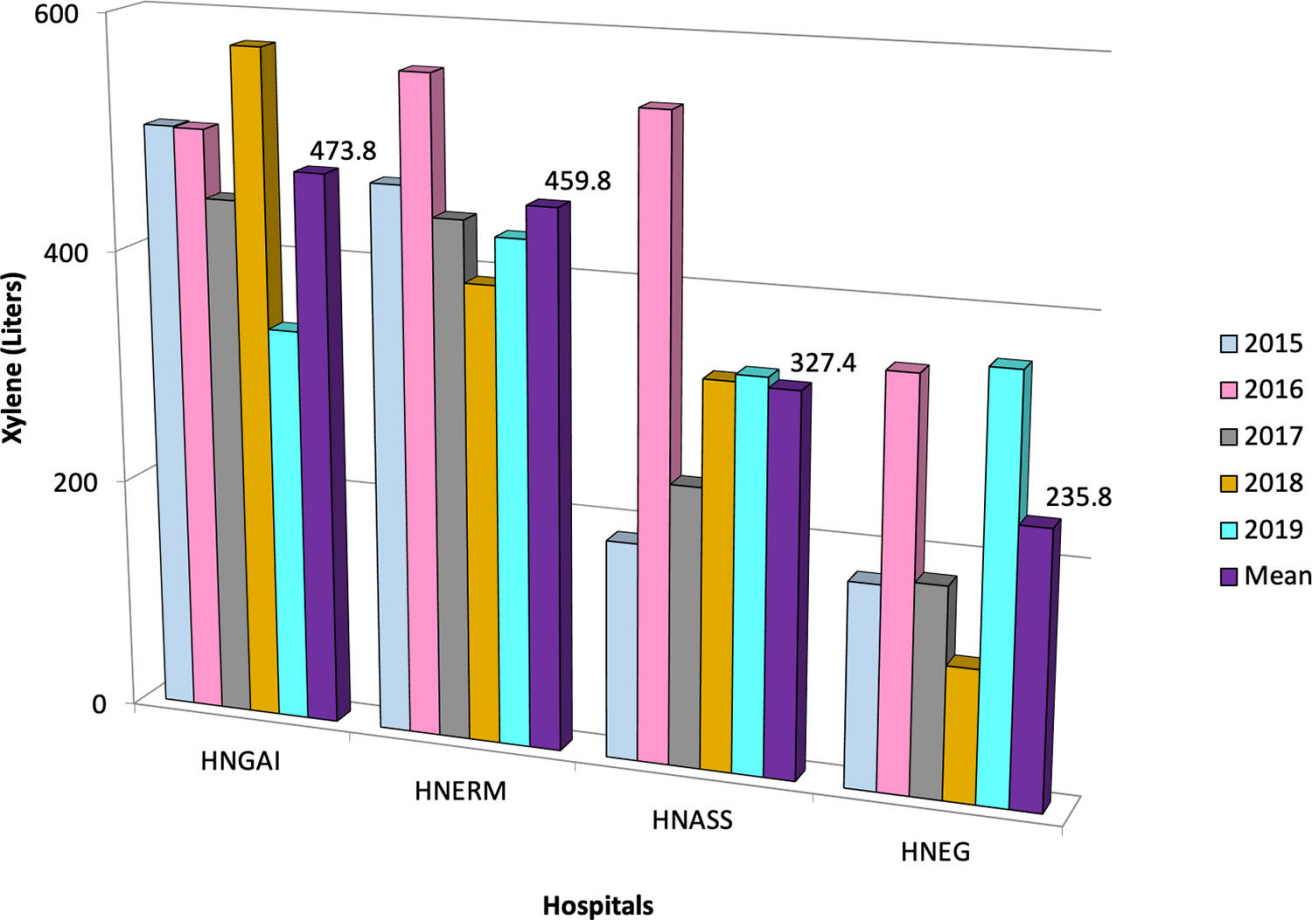
Amount of xylene used during Pap test in four Peruvian hospitals. Within the study period, the mean xylene used is shown for each year according to color. HNGAI: Hospital Nacional Guillermo Almenara Irigoyen, HNERM: Hospital Nacional Edgardo Rebagliati Martins, HNEG: Hospital Nacional de Emergencias Grau, HNASS: Hospital Nacional Alberto Sabogal Sologuren in Callao province.

We included 131,456 cytology tests in the HNGAI, 254,106 in the HNERM, 110,858 in the HNASS, and 98,478 in the HNEG. The use of xylene during for the HNGAI, HNERM, HNASS and HNEG was 2,369, 2,299, 1,637, and 1,179 liters, respectively. Overall, a total of 7,484 liters of xylene were used for Pap stains in four EsSalud hospitals during a five-year period in Lima, Peru ( Table 1). The mean rate of xylene use was 473.8 ± 88.1 liters (95%CI 396.5 to 551.1) for HNGAI, 459.8 ± 63.5 liters (95%CI 404.1 to 515.5) for HNERM, 327.4 ± 136.3 liters (95%CI 207.9 to 446.9) for the HNASS, and 235.8 ± 111.9 liters (95% CI 137.7 to 333.9) for the HNEG ( Figure 3). Using this amount of xylene in four hospitals resulted in a cost of USD 60,853 (202,068 soles).

**Table 1.** Use of xylene and costs in four EsSalud hospitals. These data were estimated according to the amount of annual Pap tests during 2015–2019.

Hospital	Year	Xylene (liters)	Cost (USD)	Cost (Soles)
HNGAI	2015	505	4106	13635
	2016	504	4098	13608
	2017	446	3627	12042
	2018	575	4676	15525
	2019	339	2756	9153
	**TOTAL**	**2369**	**19265**	**63963**
HNERM	2015	469	3814	12663
	2016	562	4570	15174
	2017	444	3610	11988
	2018	392	3187	10584
	2019	432	3513	11664
	**TOTAL**	**2299**	**18696**	**62073**
HNASS	2015	187	1520	5049
	2016	544	4424	14688
	2017	240	1951	6480
	2018	330	2683	8910
	2019	336	2732	9072
	**TOTAL**	**1637**	**13312**	**44199**
HNEG	2015	175	1423	4725
	2016	350	2846	9450
	2017	180	1463	4860
	2018	114	927	3078
	2019	360	2927	9720
	**TOTAL**	**1179**	**9588**	**31833**

Abbreviations: HNGAI: Hospital Nacional Guillermo Almenara Irigoyen, HNERM: Hospital Nacional Edgardo Rebagliati Martins, HNASS: Hospital Nacional Alberto Sabogal Sologuren, HNEG: Hospital Nacional de Emergencias Grau. Costs: 8.13 US dollars (USD) per liter (27 soles).

When estimating future costs for the five-year period from 2020 to 2025, the biggest assumption is that the use of three xylene baths in four hospitals is estimated at 9,483 liters and 77,110 USD (256040 soles). The lowest assumption for the use of three xylene baths in the next five years is estimated to be 5,485 liters and 44,590 USD (148,080 soles) (p = 0.0025) ( Table 2). For HNGAI, HNERM, HNASS and HNEG, the maximum volume of the three xylene baths used is 561.9, 523.3, 463.7 and 347.7 liters, respectively. For HNGAI, HNERM, HNASS and HNEG, the minimum volume of the three xylene baths used is 385.7, 396.3, 191.1, 123.9 liters, respectively.

**Table 2.** Quantity and, maximum and minimum costs and liters of xylene. We describe the quantities and, maximum and minimum costs estimated in this study at four EsSalud hospitals during 2015–2019 in Lima, Peru.

Hospital	Assumptions	Xylene (liters)	Cost (USD)	Cost (Soles)
HNGAI	Maximum	2809.5	22845	75855
	Mínimum	1928.5	15680	52070
HNERM	Maximum	2616.5	21275	70645
	Mínimum	1981.5	16110	53500
HNASS	Maximum	2318.5	18855	62600
	Mínimum	955.5	7765	25785
HNEG	Maximum	1738.5	14135	46940
	Mínimum	619.5	5035	16725

Abbreviations: HNGAI: Hospital Nacional Guillermo Almenara Irigoyen, HNERM: Hospital Nacional Edgardo Rebagliati Martins, HNASS: Hospital Nacional Alberto Sabogal Sologuren, HNEG: Hospital Nacional de Emergencias Grau. Costs: 8.13 US dollars (USD) per liter (27 soles).

**Figure 4.**
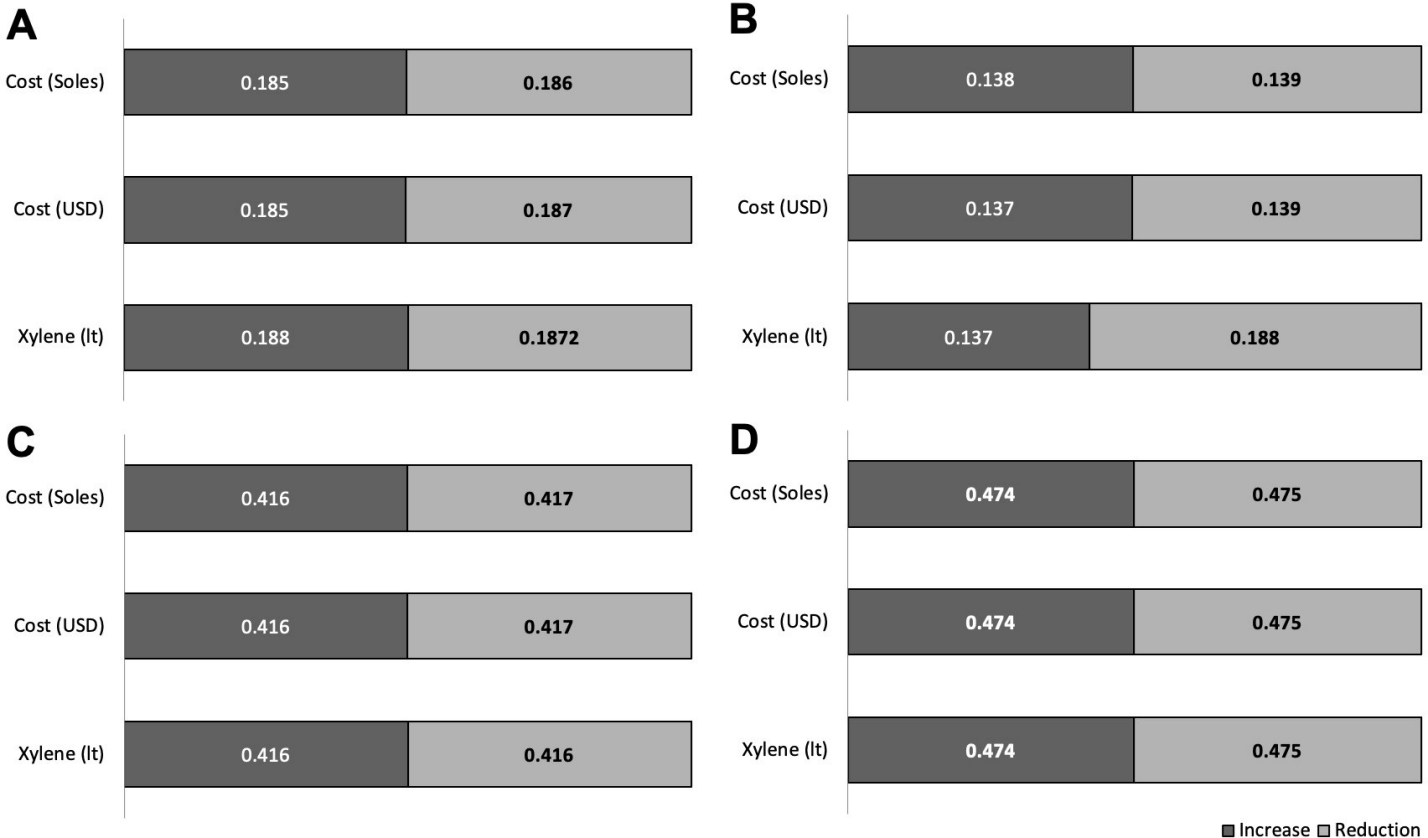
Increase or reduction (in liters and costs) of xylene in four EsSalud hospitals with the use of three xylene-baths in Pap staining. A. Hospital Nacional Guillermo Almenara Irigoyen, B. Hospital Nacional Edgardo Rebagliati Martins, C. Hospital Nacional Alberto Sabogal Sologuren, D. Hospital Nacional de Emergencias Grau. The increase indicates the maximum percentage of increase in the 2020–2025 periods in liters and costs of xylene. The reduction indicates the minimum percentage of reduction in the five-year period 2020–2025 in liters and costs of xylene.

These estimates of future costs showed increases and decreases according to the hospitals ( Figure 4). In the four hospitals, the liter and cost of xylene increased by 30.4% and 30.3% USD on average, respectively. The average reduction in the cost and liters of xylene was 25.3% liters and 24.3% USD, respectively. A difference was determined between the increases in xylene use among EsSalud hospitals (p = 0.0001).

## Discussion

We determined that xylene had a high impact during the Pap test with two baths in four EsSalud hospitals, and a dramatic increase in costs and xylene contamination in the next five years (2020–2025) with the use of three xylene baths on the Pap smear according to the recommendations of the 2018 EsSalud cytology guidelines.

In the environment, xylene can cause air pollution due to the production of toxic gases (when it is thermally decomposed), polluting water, soil, and subsoil. ^16^ Inhalation of xylene can irritate the mucous membranes of the nose and throat, and high concentrations can cause nausea, vomiting, headache, respiratory failure, cough, heart abnormalities, proteinuria, and hematuria. ^17^ Cancer, leukemia, brain tumors and tissue changes (pulmonary long-term exposure and long-term high concentration) are also related to xylene contamination. ^18^^,^^19^ Other effects include fetal kidney disease, infertility and miscarriage in children whose mothers have been in contact with xylene for a long time. ^20^

Our estimates of xylene use in hospitals indicate that 594,898 cervical smears analyzed by Pap staining use >7,000 liters of xylene every five years. The same estimation of the use and contamination by xylene can be applied in other realities such as the 243,374 smears of the Hospital Nacional Docente Madre Niño San Bartolomé in Lima during two periods [118,016 for the conventional Pap staining (2011–2013 years) and 125,358 for the prolonged Pap staining (2013–2014 years)], ^11^^,^^21^ to the 3,276,045 cervical smears of the Instituto Mexicano del Seguro Social in Mexico, ^22^ and in the 60 million cervical smears performed annually in the United States. ^23^ We can infer that in every country, cervical cancer screening will involve a large amount of xylene, which will threaten the health of laboratory workers and global environmental health.

Implementing a third xylene bath to the Pap test would undoubtedly have even more counterproductive results. As our findings indicate, there will be an increase in the overall costs of the tests (77,110 USD), the time to stain each sample, and the daily exposure of workers to higher amounts of xylene (9,483 liters). A previous study demonstrated that 37 national guidelines on prevention measures for the use of xylene in South American cytology laboratories were of little importance. ^24^ In Peru, xylene is usually stored after use until disposal. It is not reused due to the regulation of controlled reagents, therefore it bioaccumulates until its elimination as a whole. However, there is no global regulation that controls all health centers, so many hospitals eliminate xylene through the drain as well as other hospitals that massively bioaccumulate xylene until they are finally disposed of without environmental protection due to the inefficient waste management system. In this sense, under the actual conditions of cytological screening in low-and-middle-income countries, adding an additional xylene bath to the Pap stain is unsustainable and therefore should not be considered.

Currently, there are xylene substitutes that allow diagnostic activities to be carried out in the pathology laboratory in a less toxic and eco-friendly environment. These substitutes are mainly the solution of Neo-Clear (Merck, Darmstadt, Germany), Pathoclear (Biopack, Buenos Aires, Argentina), Master clear (American MasterTech Scientific, CA, USA), UltraClear™ (Avantor’s JT Baker, Deventer, Netherlands), Shandon™ (Thermo Scientific, Walthman, MA, USA), and Ottix Plus (DiapathS.pA, Martinengo, Italy), which have shown different degrees of performance and health risks. ^25^^–^^27^ Regardless of the opportunities these surrogates offer, they come with costs for Pap processing that many cytology labs cannot afford. In view of this, Eco-Pap (patent application pending) brings an alternative to ecological staining, which can reduce the number of reagents, staining time and cost without reducing test performance. ^8^^,^^12^

On the other hand, our estimated analysis model for three xylene baths shows that the cost of the Pap stain and the liters of xylene solvent are reduced. However, this assumption could not be further from reality, given that exfoliative cytology has a new stage. ^28^ This fact is partly due to the co-testing in which the Human Papilomavirus test and cytology are used as a strategy with better performance and diagnostic accuracy. ^29^ It is also because many countries, mainly low- and middle-income countries, have strategies based on exfoliative cytology due to the benefits that this technique offers and for the economic limitations that these countries face. ^30^^,^^31^

Another component that distances this assumption of reducing quantities and costs of xylene from reality is undoubtedly the alarming increase in the incidence and mortality from cervical cancer between 2012 and 2018 according to the International Agency for Research on Cancer (IARC) from 527,624 and 569,847 cases for the former, and from 265,672 to 311,365 cases for the second. ^32^

In this sense, the use of cytology as a cervical cancer strategy will continue to develop and grow around the plausibility of other techniques such as molecular biology. Therefore, if a xylene bath is added to the Pap test, the amount and cost of xylene in Peru’s EsSalud Hospitals will increase by an average of 16.6% in the next five years.

This study had limitations. 1) The analysis was conducted in four EsSalud hospitals in Lima, however, there are other hospitals that have not been included. Further studies that include other Social Security hospitals are needed; 2) This is the first study in Peru that evaluates the cost of using xylene in cervical cytology. However, due to restricted access and the limited availability of data from each hospital, the cost found has not included the time of use, the need for skilled labor, process automation, xylene bioaccumulation, waste management, and the use of xylene substitutes. These characteristics should be included in future studies; and 3) The analysis evaluated the xylene used in the Pap tests; however, it is necessary to evaluate its impact on histopathology.

## Conclusions

We demonstrated a high economic impact of xylene during the Pap test with two xylene baths in four EsSalud hospitals, in Lima, Peru. Our three-bath estimates for 2020–2025 indicate that the economic growth of xylene use is significant, which is why the 2018 EsSalud cytology guidelines are not applicable.

Nowadays, there are some alternative methods that can make Pap smears free of xylene during the clearing process, thereby becoming ecofriendly, which is beneficial to occupational health and global environmental health.

## Data availability

### Underlying data

Figshare: ‘Cost and pollution by the use of xylene in cervical cytology in four Peruvian hospitals’,

https://doi.org/10.6084/m9.figshare.14615508.v1 ^13^

This project contains the following underlying data:

• Table 1. Xylene database used in EsSalud hospitals 2015–19

Figshare: ‘Cost and pollution by the use of xylene in cervical cytology in four Peruvian hospitals’, https://doi.org/10.6084/m9.figshare.15048903.v1. ^14^

This project contains the following underlying data:

• 2018 EsSalud Cytology Guideline – Chapter 9 (English translation)

Data are available under the terms of the Creative Commons Attribution 4.0 International license (CC-BY-4.0)
