# Supplementary material for: Cost and pollution by the use of xylene in cervical cytology in four Peruvian hospitals
Source: F1000Res. 2022 Jun 8;10:848. Originally published 2021 Aug 25. [Version 3] doi: 10.12688/f1000research.52769.3 (PMC9206110; doi:10.12688/f1000research.52769.3)
Supplement: Peer review history

# Peer review: Reviewer response for version 3

**DOI:** 10.5256/f1000research.134789.r140074

**Published:** 2022-06-17

**Authors:** Nasar Yousuf Alwahaibi

**Affiliations:** 1 Allied Health Sciences Department, College of Medicine and Health Sciences, Sultan Qaboos University, Muscat, Oman

Those comments are satisfactory to me. I approve this manuscript.

Is the work clearly and accurately presented and does it cite the current literature?

Partly

If applicable, is the statistical analysis and its interpretation appropriate?

I cannot comment. A qualified statistician is required.

Are all the source data underlying the results available to ensure full reproducibility?

Partly

Is the study design appropriate and is the work technically sound?

Partly

Are the conclusions drawn adequately supported by the results?

Yes

Are sufficient details of methods and analysis provided to allow replication by others?

Yes

Reviewer Expertise:

Histopathology

I confirm that I have read this submission and believe that I have an appropriate level of expertise to confirm that it is of an acceptable scientific standard.

# Peer review: Reviewer response for version 3

**DOI:** 10.5256/f1000research.134789.r140073

**Published:** 2022-06-10

**Authors:** Alexandre Rieger, Alessandra Koehler

**Affiliations:** 1 Postgraduate Program in Health Promotion (PPGPS), University of Santa Cruz do Sul (UNISC), Santa Cruz do Sul, Brazil; 2 Institute of Basic Health Sciences, Federal University of Rio Grande do Sul (UFRGS), Porto Alegre, Brazil

I have no additional comments.

Is the work clearly and accurately presented and does it cite the current literature?

No

If applicable, is the statistical analysis and its interpretation appropriate?

I cannot comment. A qualified statistician is required.

Are all the source data underlying the results available to ensure full reproducibility?

Yes

Is the study design appropriate and is the work technically sound?

No

Are the conclusions drawn adequately supported by the results?

Yes

Are sufficient details of methods and analysis provided to allow replication by others?

Partly

Reviewer Expertise:

Molecular biology; Genetics; Genotoxicity

We confirm that we have read this submission and believe that we have an appropriate level of expertise to confirm that it is of an acceptable scientific standard.

# Peer review: Reviewer response for version 2

**DOI:** 10.5256/f1000research.122976.r135441

**Published:** 2022-05-23

**Authors:** Nasar Yousuf Alwahaibi

**Affiliations:** 1 Allied Health Sciences Department, College of Medicine and Health Sciences, Sultan Qaboos University, Muscat, Oman

The authors have responded to all comments except that xylene details are still missing.

Is the work clearly and accurately presented and does it cite the current literature?

Partly

If applicable, is the statistical analysis and its interpretation appropriate?

I cannot comment. A qualified statistician is required.

Are all the source data underlying the results available to ensure full reproducibility?

Partly

Is the study design appropriate and is the work technically sound?

Partly

Are the conclusions drawn adequately supported by the results?

Yes

Are sufficient details of methods and analysis provided to allow replication by others?

Yes

Reviewer Expertise:

Histopathology

I confirm that I have read this submission and believe that I have an appropriate level of expertise to confirm that it is of an acceptable scientific standard, however I have significant reservations, as outlined above.

# Peer review: Reviewer response for version 2

**DOI:** 10.5256/f1000research.122976.r135440

**Published:** 2022-05-11

**Authors:** Alexandre Rieger, Alessandra Koehler

**Affiliations:** 1 Postgraduate Program in Health Promotion (PPGPS), University of Santa Cruz do Sul (UNISC), Santa Cruz do Sul, Brazil; 2 Institute of Basic Health Sciences, Federal University of Rio Grande do Sul (UFRGS), Porto Alegre, Brazil

We appreciate the corrections made by the authors according to our comments.

Is the work clearly and accurately presented and does it cite the current literature?

No

If applicable, is the statistical analysis and its interpretation appropriate?

I cannot comment. A qualified statistician is required.

Are all the source data underlying the results available to ensure full reproducibility?

Yes

Is the study design appropriate and is the work technically sound?

No

Are the conclusions drawn adequately supported by the results?

Yes

Are sufficient details of methods and analysis provided to allow replication by others?

Partly

Reviewer Expertise:

Molecular biology; Genetics; Genotoxicity

We confirm that we have read this submission and believe that we have an appropriate level of expertise to confirm that it is of an acceptable scientific standard.

# Peer review: Reviewer response for version 1

**DOI:** 10.5256/f1000research.56088.r122077

**Published:** 2022-02-22

**Authors:** Alexandre Rieger, Alessandra Koehler

**Affiliations:** 1 Postgraduate Program in Health Promotion (PPGPS), University of Santa Cruz do Sul (UNISC), Santa Cruz do Sul, Brazil; 2 Institute of Basic Health Sciences, Federal University of Rio Grande do Sul (UFRGS), Porto Alegre, Brazil

The manuscript brings a relevant theme, but I believe it is not suitable for indexing, according to the following comments:

- What were the criteria for choosing the hospitals included in the study?
- A very important information is missing. What are the reasons why the 2018 EsSalud cytology guidelines recommend using three baths of xylene? I didn't find this information. I believe this is important to mention, as clearly the addition of one more xylene bath increases costs and environmental impact. Therefore, there must be a coherent reason for this recommendation, which perhaps outweighs the harm of adding one more bath.
- The cost analyzed should include factors other than the value of a liter of xylene, such as: time, need for skilled labor, possibility of automation, generation and disposal of waste.
- Substitutes for the use of xylene are suggested. It is important, however, to compare the costs and also the quality of the results, in addition to illustrating them better. This would bring more impact to the conclusion, which unfortunately did not add many scientific gains, because, if only the cost and environmental impact are compared, certainly using three xylene baths instead of two will have more impact.

Is the work clearly and accurately presented and does it cite the current literature?

No

If applicable, is the statistical analysis and its interpretation appropriate?

I cannot comment. A qualified statistician is required.

Are all the source data underlying the results available to ensure full reproducibility?

Yes

Is the study design appropriate and is the work technically sound?

No

Are the conclusions drawn adequately supported by the results?

Yes

Are sufficient details of methods and analysis provided to allow replication by others?

Partly

Reviewer Expertise:

Molecular biology; Genetics; Genotoxicity

We confirm that we have read this submission and believe that we have an appropriate level of expertise to state that we do not consider it to be of an acceptable scientific standard, for reasons outlined above.

# Response

**Published:** 2022-03-28

**Authors:** Jeel Moya-Salazar

**Affiliations:** Universidad Norbert Wiener, Peru

Dear reviewer,

 We welcome feedback. We made the following modifications in our study:

- *What were the criteria for choosing the hospitals included in the study?*
- **Response: **We include the inclusion criteria in the methodology section.
- *Very important information is missing. What are the reasons why the 2018 EsSalud cytology guidelines recommend using three baths of xylene? I didn't find this information. I believe this is important to mention, as clearly the addition of one more xylene bath increases costs and environmental impact. Therefore, there must be a coherent reason for this recommendation, which perhaps outweighs the harm of adding one more bath.*
- **Response: **We have included this information in our methods. Basically, the purpose is to improve cytology procedures, without proposing an analysis of cost-effectiveness or environmental impact of the use of xylene.

- *The cost analyzed should include factors other than the value of a liter of xylene, such as: time, need for skilled labor, possibility of automation, generation and disposal of waste. Substitutes for the use of xylene are suggested. It is important, however, to compare the costs and also the quality of the results, in addition to illustrating them better. This would bring more impact to the conclusion, which unfortunately did not add many scientific gains, because, if only the cost and environmental impact are compared, certainly using three xylene baths instead of two will have more impact.*
- **Response: **We want to do a more structured analysis, but we don't have access to the data to include more variables. Therefore, we limit our study to a cost/liter analysis as a preliminary approximation to the topic. These features are added in the limitations section.

Sincerely,

# Peer review: Reviewer response for version 1

**DOI:** 10.5256/f1000research.56088.r92919

**Published:** 2021-09-08

**Authors:** Nasar Yousuf Alwahaibi

**Affiliations:** 1 Allied Health Sciences Department, College of Medicine and Health Sciences, Sultan Qaboos University, Muscat, Oman

Although the study has academic merit, however, it lacks some important information:

- In the methodology, there are no inclusion and exclusion criteria for this retrospective study.
- Xylene details are missing.
- The manual staining for cervical cytology procedures in the laboratory (Section 9.5.1) is written in a different language but not in English. Please have a translation or a brief summary for the procedure.
- Many low and middle-income countries recycle xylene and other chemical solutions used in histopathology to overcome the high cost of those solutions as well as to provide a pollution-prevention solution for the laboratory, the discussion section does not discuss this issue.
- How do you get rid of xylene in Peru? Usually, the management of xylene is carried out by means of specific legislation created by the specific regulatory agency for this purpose.
- Please justify the choice of only those four hospitals in Peru.
- In Figure 2 and in all mentioned laboratories, there is no safety cabinet to cover the xylene containers, which is a concern for biomedical scientists working in the laboratory.
- Please note that the title for the tables should be up, and the title for the figures should be underneath the figures.

Is the work clearly and accurately presented and does it cite the current literature?

Partly

If applicable, is the statistical analysis and its interpretation appropriate?

I cannot comment. A qualified statistician is required.

Are all the source data underlying the results available to ensure full reproducibility?

Partly

Is the study design appropriate and is the work technically sound?

Partly

Are the conclusions drawn adequately supported by the results?

Yes

Are sufficient details of methods and analysis provided to allow replication by others?

Yes

Reviewer Expertise:

Histopathology

I confirm that I have read this submission and believe that I have an appropriate level of expertise to confirm that it is of an acceptable scientific standard, however I have significant reservations, as outlined above.

# Response

**Published:** 2022-03-28

**Authors:** Jeel Moya-Salazar

**Affiliations:** Universidad Norbert Wiener, Peru

Dear Reviewer, 

 We welcome feedback. We have made modifications to the study on the following points:

- *In the methodology, there are no inclusion and exclusion criteria for this retrospective study.*
- **Response: **Criteria of inclusion/exclusion have been included.

- *The manual staining for cervical cytology procedures in the laboratory (Section 9.5.1) is written in a different language but not in English. Please have a translation or a brief summary for the procedure.*
- **Response: **A short version of the procedure is included in reference 13. It's on Figshare
- *Many low and middle-income countries recycle xylene and other chemical solutions used in histopathology to overcome the high cost of those solutions as well as to provide a pollution-prevention solution for the laboratory, the discussion section does not discuss this issue.*
- **Response: **We added in the discussion.
- *How do you get rid of xylene in Peru? Usually, the management of xylene is carried out by means of specific legislation created by the specific regulatory agency for this purpose.*
- **Response: **We added in the discussion.
- *Please justify the choice of only those four hospitals in Peru.*
- **Response: **We selected four hospitals because they are the main ones of EsSalud and they were the ones that agreed to be part of the study.
- *In Figure 2 and in all mentioned laboratories, there is no safety cabinet to cover the xylene containers, which is a concern for biomedical scientists working in the laboratory.*
- **Response: **We agree with what you say, it is important to note that the four main hospitals in Lima do not yet have security booths. This is why, based on our research, we believe that adding one more xylene bath is not enough.

- *Please note that the title for the tables should be up, and the title for the figures should be underneath the figures*
- **Response: **It is an editorial issue that we already adjusted.

Sincerely
